# Association between production and reproduction parameters based on parity and breed of dairy cows in the Czech Republic

**DOI:** 10.5194/aab-67-197-2024

**Published:** 2024-04-23

**Authors:** Marek Vrhel, Jaromír Ducháček, Matúš Gašparík, Mojmír Vacek, Radim Codl, Jan Pytlík

**Affiliations:** Department of Animal Science, Faculty of Agrobiology, Food and Natural Resources, Czech University of Life Sciences Prague, Kamýcká 129, 165 00 Prague-Suchdol, Czech Republic

## Abstract

Milk production and the efficiency of dairy cow breeding are significantly influenced by reproductive factors. The purpose of our research was to examine the relationships between selected milk production and reproductive parameters. We evaluated 659 dairy cows, including 444 purebreds from the Czech Fleckvieh and Holstein breeds and 215 crossbreds. Our primary objective was to assess the impacts of breed and parity on specific milk production and reproductive parameters. The study revealed significant results regarding the interaction between certain breed groups and parity. In particular, there was a noticeable increase in milk yield with parity. Furthermore, it was also evident that the highest milk yield values were related to the milk content. Breed group H, which represents cows with a more than 50 % Holstein bloodline, had the highest values of the monitored milk content. Our findings show that first-lactation crossbred Czech Fleckvieh cows had a reduced milk yield, decreased fat, and lactose content in milk. However, they had a more favourable calving interval when compared to purebred Czech Fleckvieh and higher-parity Holstein crossbreds. Crossbred H, in comparison to C100 and C50, whether purebred or crossbred with Czech Fleckvieh, showed a relatively higher content of some milk components. The results for service periods and calving intervals were not statistically significant. The findings of this study highlight the promising potential of higher-parity Holstein crossbred cows in terms of milk yield and the advantages of lower-parity ones concerning milk contents.

## 1 Introduction

Studies from foreign publications have shown a negative relationship between high milk production and decreasing fertility, especially in cows with predominantly Holstein blood (Lehmann et al., 2016; Barzehkar et al., 2023). The research of Cabrera (2014) confirms an increasing trend in the productivity of dairy cows; however, there is a concurrent decline in reproductive parameters, as demonstrated by research conducted in Brazil (Madureira et al., 2022). The efficiency of dairy cow production depends on milk yield (Habib et al., 2020), feed cost (Hamed and Kamel, 2021), and reproduction level, which is a basic prerequisite for the sustainability of dairy farming (El-Tarabany and El-Bayoumi, 2015).

It is well-known that low fertility in dairy cattle leads to a prolonged calving interval (CalvInt), which in turn is associated with reduced annual milk yield and thus profitability of dairy farming (Nyman et al., 2018). An important factor that influences milk yield and milk composition is the length of lactation, which is determined by the length of the service period (SP) (Leitner et al., 2012). The optimal length of CalvInt is approximately an interval of 1 year for dairy cows with an average milk yield of approximately 7500 to 9500 kg of milk per lactation (Vijayakumar et al., 2017). Sehested et al. (2019) reported that high-producing dairy cows can have an extended CalvInt of up to 90 d. Additionally, the amount of milk produced per day will also increase based on the reduction in SP. Maheshwari et al. (2018) reported that cows on second and third lactations are the most productive, which is also confirmed by Bondan et al. (2018). Rajala-Schultz et al. (2011) in their work suggested that younger dairy cows have a higher proportion of fat and lactose and also a lower somatic cell count (SCC). Kargo et al. (2021) reported that the heterosis effect in Holsteins and their crossbreds significantly influenced milk production traits, which is also confirmed by Daltro et al. (2021). When examining the relationship between milk production and reproductive parameters, Knob et al. (2020) reported that reproductive parameters are significantly better in Czech Fleckvieh crossbreds compared to Holstein crossbreds due to shorter SP, i.e. the heterosis effect. Therefore, herd reproduction level indicators are directly related to milk yield and milk composition. Thus, monitoring and optimizing these parameters is very important in practice (Begalieva et al., 2022; Rodriguez et al., 2022).

Consequently, the aim of this study was to evaluate the relationships between the milk production parameters, selected reproductive parameters, and genetic background of animals. The second objective was to assess the effect of breed, parity, and their interaction on selected production and reproduction parameters.

## 2 Material and methods

### 2.1 Animals and samples

A total of 659 cows were included in this study. Of these, the breakdown was as follows: purebred Czech Fleckvieh ( n=392 ) (C100), crossbred with more than 50 % Czech Fleckvieh ( n=215 ) (C50), and crossbred with more than 50 % Holstein ( n=52 ) (H). The dairy cows were housed on three farms in the western region of the Czech Republic at an altitude of approximately 380–420 m above sea level. All the dairy cows were housed in free-stall barns, bedded with recycled solid manure or straw bedding. The cows were milked according to a standardized procedure three times daily in herringbone milking parlours. Total mixed ratios (TMRs) were composed of standard components (corn silage, clover silage, straw, hay, molasses, mineral mixture, and brewers thresher), and changes in their composition reflected the inter-period phase and actual milk production over the whole evaluation period.

### 2.2 Data collection

The evaluation included data from January to December 2018. Data on average milk yield per lactation (MY, kg), average fat in milk per lactation (F, kg), average protein in milk per lactation (P, kg), average lactose in milk per lactation (L, kg), average percentage of fat in milk (F%), average percentage of protein in milk (P%), and average percentage of lactose in milk (L%) were measured using the Pulsameter 2 (certified by ICAR, Labor- und Messgeräte GmbH, Germany). All the data mentioned were recorded and later retrieved using the FARMSOFT management and farm software as well as the Vitalimeter FA22 neck responder system for monitoring cattle locomotor activity (Farmtec a.s., Jistebnice, Czech Republic). The dataset was further supplemented with data on SP (d), CalvInt (d), and summary selection indices for the Czech Fleckvieh (Genetic Total Merit Index – GZW) and Holstein breed index (Selection Index Holstein – SIH) from the performance control database maintained by the Czech Moravian Breeders Corporation (ČMSCH; Hradištko, Czech Republic). The dataset was adjusted for outliers in selected production parameters which were removed. For a more detailed evaluation, the animals were categorized based on parity: the first lactation ( n=157 cows), second lactation ( n=185 cows), third lactation ( n=141 cows), and fourth and further lactations ( n=176 cows) were monitored.

### 2.3 Statistical analysis

Statistical evaluation was performed in SAS software version 9.4 (SAS/STAT^®^; SAS Institute Inc., Cary, NC, USA). Basic statistics were calculated with the UNIVARIATE procedure. Linear regression was performed using the REG procedure. The main evaluation was performed using the MIXED procedure. Several options for evaluating performance parameters were tested during model development. The REG procedure was used to select appropriate effects in the model equation using the STEPWISE method and the Akaike information criterion parameter. The fixed effects of parity, breed, parity × breed interaction, GZW/SIH, and random effect of the animal were selected for the model equation for the evaluation of monitored production and reproduction parameters. A detailed evaluation was performed using the Tukey–Kramer test. The results showed that the relationships between reproductive and production traits were not statistically significant. As a result, they were not included in the model equations for evaluation, nor were they included in the final model equation for the production parameters.

The following model equation was used for the evaluation:

1 $$\begin{array}{rl} y_{ijkl} & =\mu +\;{\mathrm{PAR}}_{i}+{\mathrm{BRE}}_{j}+\;{\left(\mathrm{PAR}\;\times \;\mathrm{BRE}\right)}_{ij}\\ & +b\times (\mathrm{GZW}/\mathrm{SIH})+{\mathrm{cow}}_{k}+\;e_{ijkl}, \end{array}$$

where $y_{ijkl}$ is the measured value of the dependent variables MY – milk yield per lactation (kg), F – fat in milk per lactation (kg), P – protein in milk per lactation (kg), L – lactose in milk per lactation (kg), F% – percentage of fat in milk (%), P% – percentage of protein in milk (%), L% – percentage of lactose in milk (%), SP – length of the service period (time from calving to successful insemination of the cow) (d), CalvInt – calving interval (d), μ – mean value of the dependent variable, PAR ${}_{i}$ – fixed effect of parity (where ${}_{i}=1$ , n=157 ; ${}_{i}=2$ , n=185 ; ${}_{i}=3$ , n=141 ; ${}_{i}=\geq 4$ , n=176 ), BRE ${}_{j}$ – fixed effect of the breed (where ${}_{j}=$ C100, n=392 ; ${}_{j}=$ C50, n=215 ; ${}_{j}=$ H, n=52 ), (PAR × BRE) ${}_{ij}$ – interaction between effects of lactation parity and breed (C100 × 1, n=94 ; C100 × 2, n=118 ; C100 × 3, n=72 ; C100 × 4, n=108 ; C50 × 1, n=50 ; C50 × 2, n=50 ; C50 × 3, n=54 ; C50 × 4, n=61 ; H × 1, n=13 ; H × 2, n=17 ; H × 3, n=15 ; H × 4, n=7 ), b × (GZW/SIH) the linear regression on summary selection indices, cow ${}_{k}$ the random effect of the cow ( ${}_{k}=659$ ), and $e_{ijkl}$ the random residual error. Significance levels of p<0.05 and p<0.01 were used to evaluate the differences between groups.

## 3 Results

The key findings of our study are presented in Table 1, which shows the outcomes for MY. From the table, it is evident that the H group achieved the highest MY values, with an average of 8675.77 kg ( p<0.05 ). Conversely, the C50 group showed the lowest MY values, with an average of 7576.13 kg ( p<0.05 ). The C100 group posted MY values averaging 7670.01 kg ( p<0.05 ). Table 1 also showed that the highest average values of F =357.10 , P =323.64 , and L =439.27 kg were observed in the H group ( p<0.05 ). It was also found that the H breed group had the highest average fat percentage (F% =4.17 ), while the C100 and C50 breeds had fat percentages of 3.96 % and 3.94 %, respectively. A similar trend corresponds to the results of Bouallegue et al. (2014). In contrast, breed group H also had the highest protein percentage, P%, at 3.76, while the C50 breed had the lowest percentage at 3.47. The analysis of lactose percentage showed that the C100 and C50 breed groups had the highest values, with L% at 4.96 and 4.95, respectively. In the case of the H breed, the lactose percentage was found to be 4.91. Conversely, cows from the C100 and C50 breed groups achieved shorter average SP durations, which are considered better. The difference in SP was up to 17.13 d shorter for C100 and up to 23.35ṣhorter for C50 ( p<0.05 ). Evaluation of the CalvInt parameter revealed that the lowest mean durations were also achieved by the C100 and C50 breeds.

Other widely recognized reproductive parameters were not evaluated due to a lack of data for their objective assessment or the already large number of parameters evaluated in this study. Nevertheless, interesting inverse relationships between production and reproduction parameters can still be described.

**Table 1:** Basic statistical analysis of variables in the evaluated groups of cow breeds with the UNIVARIATE procedure.

	C100				C50				H			
Variables	N	Means	SD	CV	N	Means	SD	CV	N	Means	SD	CV
MY	392	7670.01	1145.19	14.93	215	7576.13	1178.08	15.55	52	8675.77	1839.64	21.20
F	392	302.05	47.67	15.78	215	296.87	47.41	15.97	52	357.10	72.31	20.25
P	392	270.25	39.63	14.66	215	261.81	40.95	15.64	52	323.64	63.95	19.76
L	392	391.76	60.51	15.44	215	387.67	61.62	15.89	52	439.27	94.95	21.62
F%	392	3.96	0.33	8.28	215	3.94	0.30	7.55	52	4.17	0.47	11.17
P%	392	3.54	0.20	5.58	215	3.47	0.20	5.83	52	3.76	0.27	7.14
L%	392	4.96	0.15	3.10	215	4.95	0.16	3.18	52	4.91	0.14	2.88
SP	346	145.51	70.93	48.75	182	139.29	55.15	39.59	42	162.64	71.54	43.99
CalvInt	346	430.51	70.93	16.48	182	424.29	55.15	13.00	42	447.64	71.54	15.98
GZW/SIH	384	97.70	7.56	7.73	183	95.67	8.23	8.60	33	96.60	8.52	8.82

MY – milk yield per lactation (kg); F – fat in milk per lactation (kg); P – protein in milk per lactation (kg); L – lactose in milk per lactation (kg); F% – percentage of fat in milk; P% – percentage of protein in milk; L% – percentage of lactose in milk; SP – length of the service period (d); CalvInt – calving interval (d); GZW/SIH – summary selection indices for the Czech Fleckvieh (GZW) and Holstein breed index (SIH) from the performance control database maintained by the Czech Moravian Breeders Corporation; C100 – purebred Czech Fleckvieh; C50 – crossbred with more than 50 % Czech Fleckvieh; H – crossbreds with more than 50 % Holstein blood; N – number of observations; SD – standard deviation; CV – coefficient of variation (%).

Notably, the effects of parity, breed, and their combined interaction within the model had significant impacts on the evaluated parameters. Table 2 presents the results from the MIXED procedure, examining the effects of parity, breed, and their interaction of breed and parity on the monitored production and reproduction parameters. The individual effects of parity and breed often paralleled the trends observed in the parity × breed interaction effect. Therefore, in the subsequent sections, only the interaction results are discussed. The results of our study, as shown in Table 2, indicate the lowest values for MY as 6731.58 and 6914.37 kg, F as 258.94 and 268.72 kg, P as 232.79 and 246.01 kg, L as 352.31 and 365.53 kg, and F% as 3.87 % and 3.92 %. These values were achieved by the Czech Fleckvieh breed group (C100), including crossbreds with a majority share (C50) during their first parity ( p<0.05 ). On the other hand, the lactose percentages, L% =5.04% and L% =5.06% , reached their highest values ( p<0.05 ). Our results indicate that the breed group with a majority of Holsteins had the highest values of MY, F, P, L, F%, and P%, regardless of parity ( p<0.05 ). The highest MY value, 9602.57 kg, was recorded for the oldest dairy cows of the H breed ( p<0.01 ). Additionally, the highest values for SP and CalvInt were achieved by the H cows in their fourth or greater parity, while the lowest values were found in the C50 breed during their fourth or greater parity as well as the C50 breed during their third parity. In the next phase of our investigation, we delved into the connection between SP and production parameters as well as their association with the GZW or SIH values. Through linear regression, we confirmed the commonly assumed functioning of these indices. Specifically, a 1 d extension in SP directly translates to an increase in MY by 1029 kg, an increase in fat F by 0.043 kg, an increase in protein P by 0.053 kg, and slight shifts in F% and L% by 0.0004 % and 0.0003 %, respectively, with statistical significance at the p<0.05 level.

**Table 2:** Evaluation of selected production and reproduction parameters (LSM ± SELSM) by using the MIXED procedure in terms of parity, breed, and parity–breed interaction.

Parity		MY	F	P	L	F%	P%	L%	SP	CalvInt
1		$7074.91\pm 168.81^{A}$	$287.54\pm 6.62^{A}$	$256.28\pm 5.87^{A}$	$368.84\pm 9.04^{A}$	4.11±0.05	$3.64\pm 0.03^{A}$	$5.02\pm 0.02^{A,a}$	160.52±10.41	445.52±10.41
2		$8121.38\pm 125.84^{B}$	$321.75\pm 4.94^{B,a}$	$294.34\pm 4.37^{B}$	$415.81\pm 6.74^{B}$	4.01±0.04	$3.64\pm 0.02^{A}$	$4.96\pm 0.02^{C,b}$	148.37±8.00	433.37±8.00
3		$8544.63\pm 130.57^{B}$	$340.77\pm 5.12^{B,b}$	$300.02\pm 4.54^{B}$	$431.50\pm 7.00^{B}$	4.00±0.04	$3.52\pm 0.02^{B}$	$4.91\pm 0.02^{B,c}$	142.97±8.68	427.97±8.68
≥4		$8392.56\pm 194.95^{B}$	$329.83\pm 7.65^{B}$	$291.67\pm 6.78^{B}$	$420.36\pm 10.44^{B}$	3.96±0.06	$3.49\pm 0.03^{B}$	$4.83\pm 0.02^{B,D,d}$	189.01±16.41	474.01±16.41
Breed										
	C100	$7675.09\pm 57.70^{A}$	$301.02\pm 2.26^{A}$	$269.75\pm 2.01^{A}$	$391.81\pm 3.09^{A}$	$3.94\pm 0.02^{A}$	$3.53\pm 0.01^{A}$	$4.95\pm 0.01^{a}$	$146.28\pm 3.75^{a}$	$431.28\pm 3.75^{a}$
	C50	$7613.30\pm 84.16^{A}$	$298.63\pm 3.30^{A}$	$262.94\pm 2.93^{A}$	$390.22\pm 4.51^{A}$	$3.94\pm 0.02^{A}$	$3.47\pm 0.01^{B,C}$	$4.95\pm 0.01^{a}$	$142.47\pm 5.53^{A}$	$427.47\pm 5.53^{A}$
	H	$8811.71\pm 213.11^{B}$	$360.27\pm 8.36^{B}$	$324.04\pm 7.41^{B}$	$445.35\pm 11.41^{B}$	$4.18\pm 0.06^{B}$	$3.72\pm 0.04^{B,D}$	$4.88\pm 0.03^{b}$	$191.90\pm 15.69^{B,b}$	$476.90\pm 15.69^{B,b}$
Parity × breed										
1	C100	$6914.37\pm 115.86^{A}$	$268.72\pm 4.54^{A,a}$	$246.01\pm 4.03^{A}$	$365.53\pm 6.21^{A,a}$	$3.92\pm 0.03^{A}$	$3.58\pm 0.02^{A}$	$5.06\pm 0.01^{A,a}$	149.66±7.23	434.66±7.23
1	C50	$6731.58\pm 196.43^{A,a}$	$258.94\pm 7.71^{A}$	$232.79\pm 6.83^{A,a}$	$352.31\pm 10.52^{A,a}$	$3.87\pm 0.06^{A}$	$3.47\pm 0.03^{A}$	$5.04\pm 0.02^{C,c}$	150.85±13.00	435.85±13.02
1	H	$7578.77\pm 453.17^{c}$	$334.97\pm 17.78^{B,b}$	$290.04\pm 15.75^{b}$	388.66±24.27	$4.54\pm 0.13^{B}$	$3.89\pm 0.08^{B,C,a}$	4.97±0.05	181.07±27.53	466.07±27.53
2	C100	$7908.10\pm 103.76^{B,C}$	$315.51\pm 4.07^{B,C}$	$282.08\pm 3.61^{B,C}$	$406.16\pm 5.56^{B,C}$	$4.00\pm 0.03^{A}$	$3.59\pm 0.02^{E,b}$	$4.99\pm 0.01^{E,b}$	145.49±6.53	430.49±6.53
2	C50	$7872.20\pm 171.49^{B,C}$	$309.24\pm 6.73^{B,C}$	$273.48\pm 5.96^{B,C}$	$410.63\pm 9.19^{B,c}$	$3.96\pm 0.05^{A}$	$3.50\pm 0.03^{D,G}$	4.98±0.02	154.11±11.10	439.11±11.10
2	H	$8583.85\pm 320.18^{B}$	$340.49\pm 12.56^{B}$	$327.44\pm 11.13^{B,D,E,c}$	$430.63\pm 17.15^{b}$	4.05±0.09	$3.85\pm 0.06^{B,F,H}$	$4.90\pm 0.04^{B,E}$	145.50±20.34	430.50±20.34
3	C100	$7958.70\pm 132.57^{B,C}$	$309.17\pm 5.20^{B,C}$	$275.28\pm 4.61^{B,F,G}$	$401.21\pm 7.10^{B,C}$	$3.90\pm 0.04^{A}$	$3.48\pm 0.02^{D,G,a}$	$4.92\pm 0.02^{B,D,a}$	142.88±8.63	427.88±8.63
3	C50	$8193.54\pm 156.60^{B,c}$	$328.20\pm 6.14^{B,C,c}$	$283.70\pm 5.44^{B,G,d}$	$416.50\pm 8.39^{B}$	$4.02\pm 0.05^{A}$	$3.47\pm 0.03^{D,G,a}$	$4.94\pm 0.02^{B,G,d,e}$	131.96±10.04	416.96±10.04
3	H	$9481.65\pm 334.72^{B,D,d}$	$384.94\pm 13.13^{B,D}$	$341.08\pm 11.63^{B,D,H}$	$476.81\pm 17.93^{B,D,d}$	4.08±0.10	3.62±0.06	$4.87\pm 0.04^{B,d}$	154.08±22.46	439.08±22.46
≥4	C100	$7919.21\pm 109.47^{B,C}$	$310.68\pm 4.29^{B,C}$	$275.61\pm 3.81^{B,F,G}$	$394.33\pm 5.86^{C,b}$	$3.93\pm 0.03^{A}$	$3.49\pm 0.02^{D,G,a}$	$4.85\pm 0.01^{B,D,F,f}$	147.10±7.58	432.10±7.58
≥4	C50	$7655.89\pm 147.54^{B,C,b}$	$298.13\pm 5.79^{B,C,d}$	$261.78\pm 5.13^{F,G,I,b}$	$381.45\pm 7.90^{C,e}$	$3.91\pm 0.04^{A}$	$3.44\pm 0.03^{B,D,F,G}$	$4.84\pm 0.02^{B,D,F,H}$	132.96±10.03	417.96±10.03
≥4	H	$9602.57\pm 554.33^{B,a}$	$380.69\pm 21.74^{B,c}$	$337.62\pm 19.27^{B,J}$	$485.31\pm 29.69^{B,f}$	4.04±0.16	3.54±0.10	$4.78\pm 0.07^{B,d}$	286.97±47.66	571.97±47.66

LSM – least-squared means; SELSM – standard error of the least-squared means; MY – milk yield per lactation (kg); F – fat in milk per lactation (kg); P – protein in milk per lactation (kg); L – lactose in milk per lactation (kg); F% – percentage of fat in milk; P% – percentage of protein in milk; L% – percentage of lactose in milk; SP – length of the service period (d); CalvInt – calving interval (d); parity – order of lactation; C100 – purebred Czech Fleckvieh; C50 – crossbreds with more than 50 % Czech Fleckvieh; H – crossbreds with more than 50 % Holstein blood; A–B, C–D, E–F, G–H, I–J – relationships between letters mean statistical significance within columns for given effects ( p<0.01 ); a–b, c–d, e–f – relationships between letters mean statistical significance ( p<0.05 ).

**Figure 1:**
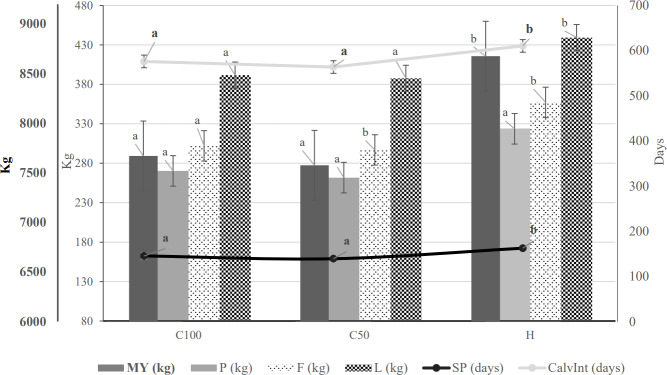
Graphical representation of the results of the selected production and reproduction parameters. MY – milk yield per lactation (kg); P – protein in milk per lactation (kg); F – fat in milk per lactation (kg); L – lactose in milk per lactation (kg); SP – length of the service period (d); CalvInt – calving interval (d); C100 – purebred Czech Fleckvieh; C50 – crossbreds with more than 50 % Czech Fleckvieh; H – crossbreds with more than 50 % Holstein blood. Different letters (a, b) within columns for a given parameter indicate statistical significance at p<0.05 .

The results of the evaluated parameters are illustrated in Fig. 1 for enhanced clarity and understanding. This figure showcases the outcomes for MY, F, P, and L at a significance level of p<0.05 . From the figure, it is evident that the H breed group surpasses both the C100 and C50 groups in terms of MY, P, F, and L at a significance level of p<0.05 . However, when it comes to the length of SP and CalvInt, both the C100 and C50 groups show shorter durations. On further comparison between the purebred group (C100) and the crossbreds (C50), C100 demonstrated better values for production parameters like MY, P, F, and L (kg). In contrast, the C50 group showed the shortest SP and CalvInt (d) at a significance level of p<0.05 . To provide a thorough analysis of these parameters, we considered both production and reproductive parameters. The results derived from our model equation turned out to be statistically significant for the majority of the parameters evaluated, with significance levels ranging from p<0.01 to 0.05. The other commonly used reproductive parameters were not evaluated due to a lack of data for their objective evaluation or due to the already large number of parameters evaluated in this study. None of the results for SP and CalvInt is statistically significant. Nevertheless, interesting inverse relationships between production and reproduction parameters can still be described.

The results of the correlation analysis for the monitored parameters are presented in Table 3. From this table, it is evident that the correlation of SP with most parameters is statistically insignificant, except for a strong positive correlation with CalvInt. This is expected, as the length of SP significantly influences the duration of CalvInt. Additionally, negative correlations were observed with L% and GZW/SIH, with values of -0.119 and -0.108 , respectively ( p<0.01 ). Parity showed a weak to moderate correlation with most parameters, except for a strong negative correlation with L% at a value of -0.515 ( p<0.01 ). A very strong correlation was observed between L and MY, registering at 0.965, and between F and P, with a value of 0.885 ( p<0.01 ). There was also a moderate correlation between F% and P% with a value of 0.468. The majority of the correlations between the parameters are positive, with a few exceptions such as the correlation between parity and L%.

**Table 3:** Correlation analysis between monitored production and reproduction parameters.

Monitored	F	P	L	F%	P%	L%	SP	CalvInt	Parity	GZW/SIH
parameters										
MY	0.864 ${}^{**}$	0.931 ${}^{**}$	0.965 ${}^{**}$	$-0.160^{**}$	$-0.123^{**}$	0.003 ${}^{\mathrm{NS}}$	0.055 ${}^{\mathrm{NS}}$	0.055 ${}^{\mathrm{NS}}$	0.200 ${}^{**}$	0.181 ${}^{**}$
F		0.885 ${}^{**}$	0.831 ${}^{**}$	0.350 ${}^{**}$	0.108 ${}^{**}$	0.010 ${}^{\mathrm{NS}}$	0.056 ${}^{\mathrm{NS}}$	0.056 ${}^{\mathrm{NS}}$	0.172 ${}^{**}$	0.254 ${}^{**}$
P			0.898 ${}^{**}$	0.014 ${}^{\mathrm{NS}}$	0.242 ${}^{**}$	0.015 ${}^{\mathrm{NS}}$	0.079 ${}^{\mathrm{NS}}$	0.079 ${}^{\mathrm{NS}}$	0.132 ${}^{**}$	0.204 ${}^{**}$
L				$-0.152^{**}$	$-0.112^{**}$	0.209 ${}^{**}$	0.054 ${}^{\mathrm{NS}}$	0.054 ${}^{\mathrm{NS}}$	0.070 ${}^{\mathrm{NS}}$	0.239 ${}^{**}$
F%					0.468 ${}^{**}$	0.028 ${}^{\mathrm{NS}}$	$-0.002^{\mathrm{NS}}$	$-0.002^{\mathrm{NS}}$	$-0.050^{\mathrm{NS}}$	0.160 ${}^{**}$
P%						0.032 ${}^{\mathrm{NS}}$	0.065 ${}^{\mathrm{NS}}$	0.065 ${}^{\mathrm{NS}}$	$-0.177^{**}$	0.070 ${}^{\mathrm{NS}}$
L%							$-0.119^{**}$	$-0.119^{**}$	$-0.515^{**}$	0.252 ${}^{**}$
SP								1.000 ${}^{**}$	$-0.006^{\mathrm{NS}}$	$-0.108^{**}$
CalvInt									$-0.006^{\mathrm{NS}}$	$-0.108^{*}$
Parity										$-0.260^{**}$

MY – milk yield per lactation (kg); F – fat in milk per lactation (kg); P – protein in milk per lactation (kg); L – lactose in milk per lactation (kg); F% – percentage of fat in milk; P% – percentage of protein in milk; L% – percentage of lactose in milk; SP – length of the service period (d); CalvInt – calving interval (d); parity – order of lactation; GZW/SIH – summary selection indices for the Czech Fleckvieh (GZW) and Holstein breed index (SIH), sourced from the performance control database maintained by the Czech Moravian Breeders Corporation; $p<0.01{=}^{**}$ ; $p<0.05{=}^{*}$ ; NS – no significance.

## 4 Discussion

In our work, we evaluated the association between selected productive parameters and parity across three dairy cow breed groups. Our findings highlighted variations in certain reproductive and production parameters between varying parities and three distinct cow breed groups (C100, C50, H) raised in the same region of western Bohemia. The results indicated that younger cows produced a lower milk yield. This finding aligns with Vrhel et al. (2021), who asserted that cows are not fully mature during their first lactation. When assessing production parameters, we observed that cows in their earlier lactations produced lower amounts of F, P, and L in milk. This observation is similarly made by Grayaa et al. (2019). The primiparous cows of the H breed exhibited relatively elevated levels of specific milk components when compared to those in the C100 and C50 breeds, with the exception of L%. This observation aligns with findings presented by Puppel et al. (2018). These authors further observed in their work on the Czech Fleckvieh breed that cows with a higher percentage of components in milk have a lower MY. In the case of H crossbred cows at second lactation, we found a relatively high percentage of components, the shortest CalvInt and SP, but the lowest MY. This corresponded to the results of the study by Ozdemir et al. (2018). The level of lactation persistency might have also influenced milk components (Brito et al., 2021). Wahinya et al. (2020) in their work focused on the selection of cows to improve lactation persistency and thus achieve maximum milk yield. However, at the same time, non-genetic factors affected performance parameters of dairy cows, as noted by Boujenane and Draga (2021). Evaluating CalvInt and SP, the highest values were found in H cows at fourth and further lactations, consistent with the work of Dalcq et al. (2017). These authors suggested that the significant increase in CalvInt and SP might have been due to the length of lactation. Coffey et al. (2016) further suggested that the heterosis effect of crossbreds improves cow reproduction, which could also have explained our non-significant findings in the evaluation. We observed an increase in milk production when longer SP-selected parameters were used. This was also confirmed by Mecitoglu (2022) and O'Hara et al. (2020). In contrast, Syrůček et al. (2017) suggested that extending SP will reduce milk production per cow per year. The results indicate a relationship between the GZW/SIH breeding indexes and the production parameters. An increase in the index value affected most of the evaluated production and reproduction parameters, as found in research by Costa et al. (2019). Thus, in general, the correctness of using the GZW/SIH in dairy cattle breeding could be suggested.

The prolongation of CalvInt can have a more negative impact on breeding in primiparous cows. On the other hand, Niozas et al. (2019) suggested that prolonging lactation can improve the reproductive performance of high-producing dairy cows. Our study showed that H dairy cows had longer CalvInt and SP and produced more milk over the entire lactation length, regardless of parity. A similar result in Danish farms was also described by Lehmann et al. (2016), who reported that milk yield in a descending lactation phase decreased on second and further lactations. The findings for primiparous cows in our work revealed that the C100 and C50 primiparous cows had a lower reproductive performance and MY compared to older cows of the same breed, which aligns with Okuyucu et al. (2018). Daltro et al. (2021) further added that these differences might have been due to reproductive management or the heterosis effect. Thus, our results suggest that high MY and long CalvInt or SP of dairy cows, irrespective of parity, are not optimal in terms of annual MY and hence breeding economics, as confirmed in the work of Vijayakumar et al. (2017). Furthermore, Rethmeier et al. (2019) found that deteriorating fertility could be compensated for to some extent by improving environmental factors such as higher levels of nutrition, optimizing stall designs from a welfare perspective, and employing modern technologies for monitoring the herd or individual animals. An interesting result was found in H crossbreds, which showed the greatest variability in MY, CalvInt, and SP across lactations. First- and second-lactation cows had relatively low SP and CalvInt, while third- and further-lactation cows had the longest SP and CalvInt. Similar observations were reported by Nasr et al. (2021). A significant result of our work was found in H cows on second lactation. These cows achieved the shortest CalvInt and SP and the lowest MY while on their third and further lactations. They achieved the longest CalvInt and SP but the highest MY. This may be related to the heterosis effect as suggested by Kargo et al. (2021). They further observed that there was a significant heterosis effect on both milk production and reproductive parameters.

## 5 Conclusions

This study confirms the importance of monitoring and evaluating the relationships between reproduction and production parameters. As mentioned in the results of our study, differences between breeds and parities can significantly influence breeding management and the treatment of individual dairy cows. The results from the linear regression analysis suggest interdependence between the evaluated parameters and highlight the importance of monitoring them. Breeding, based on combinations of breed values in selection indices, has proven to be effective and beneficial. Our study has shown that younger C50 cows have lower MY, F, P, and L and shorter CalvInt and SP compared to C100 or H cows on higher lactations. Thus, it is important to pay more attention to the influences that negatively affect their proper management. We also found that H cows on their second lactation have relatively better F, P, and L in milk compared to C100 cows. In terms of milk volume, older H cows performed better in our study, while the primiparous H crossbreds had a better percentage of F%, P%, and L%. This should be considered in reproduction and feeding management. The findings of our study suggest that more attention should be paid in the context of the heterosis effect in C50 and H crossbreds in order to maximize the production and reproductive potential of cows. These results could be a valuable foundation for further scientific research that would extend the results to more breeds and more reproductive parameters.

## Data Availability

The dataset for the study was the Association between production and reproduction parameters based on parity and breed of dairy cows in the Czech Republic (original data) (Mendeley data): 10.17632/6vy2tvv7jn.1 (Vrhel et al., 2024).
